# The process of prioritization of non-communicable diseases in the global health policy arena

**DOI:** 10.1093/heapol/czz043

**Published:** 2019-06-14

**Authors:** Olivia Heller, Claire Somerville, L Suzanne Suggs, Sarah Lachat, Julianne Piper, Nathaly Aya Pastrana, Jorge C Correia, J Jaime Miranda, David Beran

**Affiliations:** 1 Division of Tropical and Humanitarian Medicine, Geneva University Hospitals and University of Geneva, Rue Gabrielle-Perret-Gentil 4, Geneva, Switzerland; 2 Gender Centre, Graduate Institute of International and Development Studies, Ch. Eugène-Rigot 2, Geneva, Switzerland; 3 BeCHANGE Research Group, Institute of Public Communication, Università della Svizzera italiana, Via G. Buffi 13, Lugano CH, Switzerland; 4 Graduate Institute of International and Development Studies, Ch. Eugène-Rigot 2, Geneva, Switzerland; 5 CRONICAS Centre of Excellence in Chronic Diseases, Universidad Peruana Cayetano Heredia, Universidad Peruana Cayetano Heredia, Av. Armendariz 445, Miraflores, Lima 18, Peru; 6 School of Medicine, Universidad Peruana Cayetano Heredia, Av. Armendariz 445, Miraflores, Lima 18, Peru

**Keywords:** Non-communicable diseases, policy, global health, chronic diseases, low- and middle-income countries

## Abstract

Although non-communicable diseases (NCDs) are the leading cause of morbidity and mortality worldwide, the global policy response has not been commensurate with their health, economic and social burden. This study examined factors facilitating and hampering the prioritization of NCDs on the United Nations (UN) health agenda. Shiffman and Smith’s (Generation of political priority for global health initiatives: a framework and case study of maternal mortality. *The Lancet* 370: 1370–9.) political priority framework served as a structure for analysis of a review of NCD policy documents identified through the World Health Organization’s (WHO) NCD Global Action Plan 2013–20, and complemented by 11 semi-structured interviews with key informants from different sectors. The results show that a cohesive policy community exists, and leaders are present, however, actor power does not extend beyond the health sector and the role of guiding institutions and civil society have only recently gained momentum. The framing of NCDs as four risk factors and four diseases does not necessarily resonate with experts from the larger policy community, but the economic argument seems to have enabled some traction to be gained. While many policy windows have occurred, their impact has been limited by the institutional constraints of the WHO. Credible indicators and effective interventions exist, but their applicability globally, especially in low- and middle-income countries, is questionable. To be effective, the NCD movement needs to expand beyond global health experts, foster civil society and develop a broader and more inclusive global governance structure. Applying the Shiffman and Smith framework for NCDs enabled different elements of how NCDs were able to get on the UN policy agenda to be disentangled. Much work has been done to frame the challenges and solutions, but implementation processes and their applicability remain challenging globally. NCD responses need to be adapted to local contexts, focus sufficiently on both prevention and management of disease, and have a stronger global governance structure.

**Key Messages**

- Grouping of non-communicable diseases (NCDs) into one disease category improved traction in their prioritization.
- The economic framing, presenting rising costs and burden, was key to advance the issue on the global health policy agenda.
- The network of NCD experts remains in the health realm and has not successfully expanded their political coalition to other stakeholders whose engagement is required.
- There is the need for different policy approaches for NCD prevention vs NCD management and care.

## Introduction

Non-communicable diseases (NCDs) have featured on the World Health Organization (WHO) agenda since the early 1980s (World Health Organization, 1981) and have been on the rise globally (Naghavi *et al.*, 2017). Until September 2018, WHO included four main diseases encompassed by the NCD category, namely cardiovascular diseases (CVD), chronic respiratory diseases (CRD), diabetes and cancers with mental health being added at this time. In 2016, NCDs accounted for 41 million deaths or 71% of global mortality (World Health Organization, 2018a). CVD represented 31%, cancers 16%, CRD 7%, diabetes 3% and other NCDs 15% of global deaths. Low- and middle-income countries (LMIC) accounted for 78% of all NCD deaths and 85% of premature adult NCD deaths worldwide, with the risk of dying from an NCD being double that for an adult in a high-income country (Naghavi *et al.*, 2017).

Despite being the leading cause of morbidity and mortality worldwide, NCDs have not received the same political or financial attention from the global health community as other conditions, such as HIV/AIDS (Beaglehole *et al.*, 2011b; Horton, 2015; Dieleman *et al.*, 2016) with this group of diseases only receiving 1.7% of the US$37.6 billion in development assistance for health (Dieleman *et al.*, 2016). NCDs were absent from the Millennium Development Goals (MDGs) (Mamudu *et al.*, 2011), but are now included in the Sustainable Development Goals (SDG) (United Nations, 2016) with the WHO having developed the Global Action Plan for the prevention and control of NCDs 2013–20 (GAP) (World Health Organization, 2013) as a result of a United Nations High-Level Meeting (UNHLM) held in 2011 (UNGA, 2012). Both the SDGs and the GAP provide specific goals and targets, among others the reduction of premature mortality from NCDs by one-third by the year 2030. Both national and global responses are needed, including political will and funding (Horton and Sargent, 2018; Nugent *et al.*, 2018).

The process of prioritizing health issues at global level is complex and deeply political. A number of analytical frameworks can be used to identify factors that shape political prioritization and policy-responses (Walt *et al.*, 2008; Gilson *et al.*, 2018). It has long been recognized that health policies are formed through complex inter-relationships of content, context, process and actors (Buse *et al.*, 2012). This model first proposed by Walt and Gilson (1994) to systematically evaluate different factors that impact policy, was built on by Shiffman and Smith (2007) with the introduction of further concepts using four categories (1) the power of actors involved, (2) the ideas they use to portray the issue, (3) the nature of the political contexts in which they operate and (4) characteristics of the issue itself. (Table 1) This model has shown relevance across many areas of health such as addressing alcohol harm (Schmitz, 2016), maternal mortality (Shiffman and Smith, 2007), pneumonia (Berlan, 2016), surgically treatable conditions (Shawar *et al.*, 2015), tobacco use (Gneiting, 2016), urban health and tuberculosis (Quissell and Walt, 2016; Shawar and Crane, 2017), but has not been used to analyse how NCDs were included in the global health policy at the UNHLM and WHO.

**Table 1:** The four categories of the Shiffman and Smith model

Element from model	Description
Actor power Policy community cohesion Leadership Guiding institutions Civil society mobilization	Actor power is defined as, ‘the strength of the individuals and organizations concerned with the issue’. There are four factors, namely policy community cohesion; leadership; guiding institutions; and civil society mobilization. The unity between the various actors involved in the issue is described Identified champions for the cause capable of uniting the policy community. Guiding institutions have the mandate to lead the initiative The extent to which international and national political authorities are pressed from grassroots organization to tackle the issue at the global level.
Ideas 5. Internal frame 6. External frame	The way that the issue is portrayed and understood by those involved. 5.The internal frame seeks to grasp the level of agreement within the policy community of causes and solutions. 6.The external frame looks at how this internal frame is endorsed or not by political leaders through action.
Political contexts 7. Policy windows 8. Global governance structure	Political contexts are the overall environment in which the actors operate. It is composed of two elements. 7.Policy windows are given moments in time when actors can influence decision-makers as the policy environment is prepared to address this issue. These are, e.g. following a given political event, major disaster, etc. 8.The global governance structure is the existence of a ‘platform’ to allow for ‘effective collective action’ to enforce a set of norms.
Issue characteristics 9. Credible indicators 10. Severity 11. Effective interventions	This component describes the different elements of the issue’s nature. 9.Looking at factors as whether or not there are clear measures that show the severity of the problem and that also define how improvements are measured; 10. The magnitude of the issue vs other problems; 11. Whether or not cost-effective, evidence based, easy to achieve and low-cost measures exist and if these can be easily understood by policymakers and implemented.

This study aims to understand how NCDs gained traction on the global health agenda, which resulted in their inclusion in the 2011 UNHLM and the development of the GAP by WHO, using the Shiffman and Smith (2007) framework to identify the factors facilitating or hampering this prioritization.

## Methods

**Figure 1.**
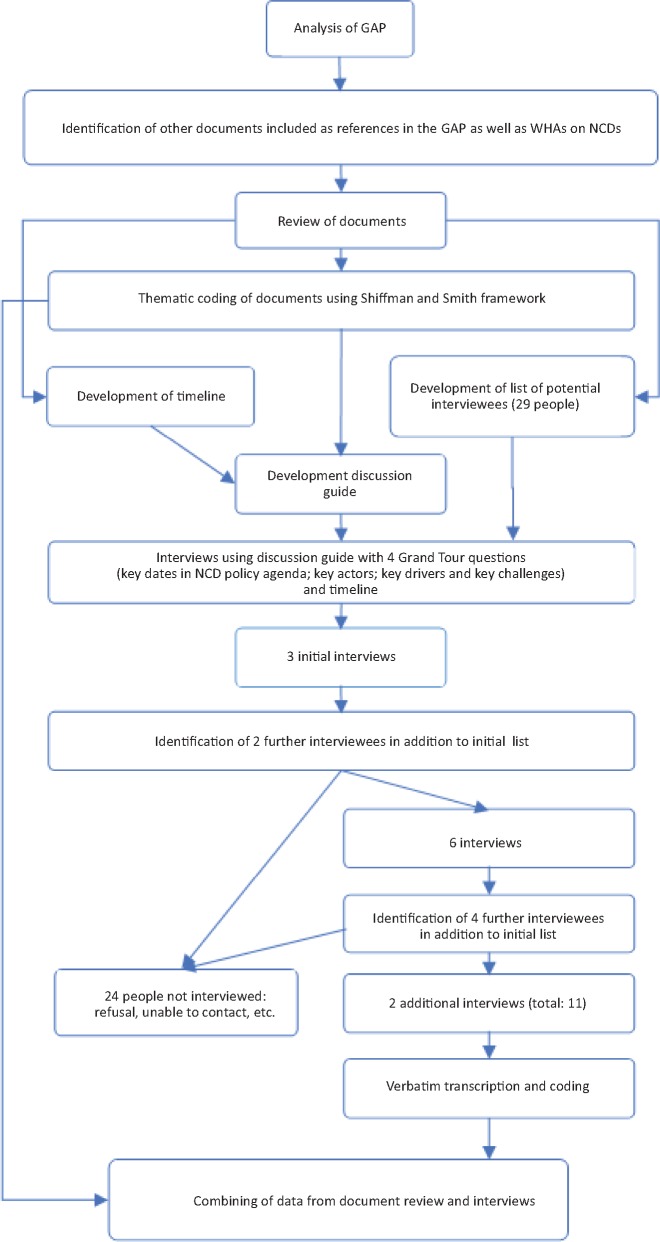
Methodological approach.

A similar approach to other researchers using the Shiffman and Smith (2007) model was adopted combining document reviews and key informant interviews using a case study method (Shiffman and Smith, 2007; Shawar *et al.*, 2015; Berlan, 2016; Gneiting, 2016; Quissell and Walt, 2016; Schmitz, 2016; Shawar and Crane, 2017) with a retrospective approach (Parsons, 1995). The methods are detailed in Figure 1. As the aim was to identify factors leading to the inclusion of NCDs in the 2011 UNHLM, with the WHO GAP (World Health Organization, 2013) being used as a starting point for the review of the literature as this was seen as the result of the overall process of prioritization of NCDs. References included in the GAP, NCD-related World Health Assembly (WHA) resolutions and documents mentioned by interviewees were analysed using the Shiffman and Smith framework. These documents and resolutions provided information to develop a timeline displaying the trajectory of key dates in the NCDs policy agenda, which was referenced during in-depth interviews and drawn upon for the analysis of the different components of the Shiffman and Smith (2007) framework.

The policy analysis was complemented with key informant interviews. Based on the document review, a list of key actors and potential interviewees was developed. Three key informants (two from the Civil Society, one from WHO/Government) were purposively contacted due to their important roles in setting the NCD agenda within the UN system. Further interviewees were identified through snowball sampling. The interviews followed a discussion guide with four sets of grand tour questions (Spradley, 1979) included in Figure 1. Interviews were conducted in person or by phone between February and September 2017 and lasted an average of 1 h. Each interview was audio-recorded. All interviews were transcribed verbatim and anonymized. Verbatim transcriptions were iteratively coded and clustered together with the document review using the Shiffman and Smith (2007) framework meta-themes, for final analysis and interpretation. This study was approved by the Commission Cantonale d'éthique de la recherche Genève in 2016.

## Results

**Figure 2.**
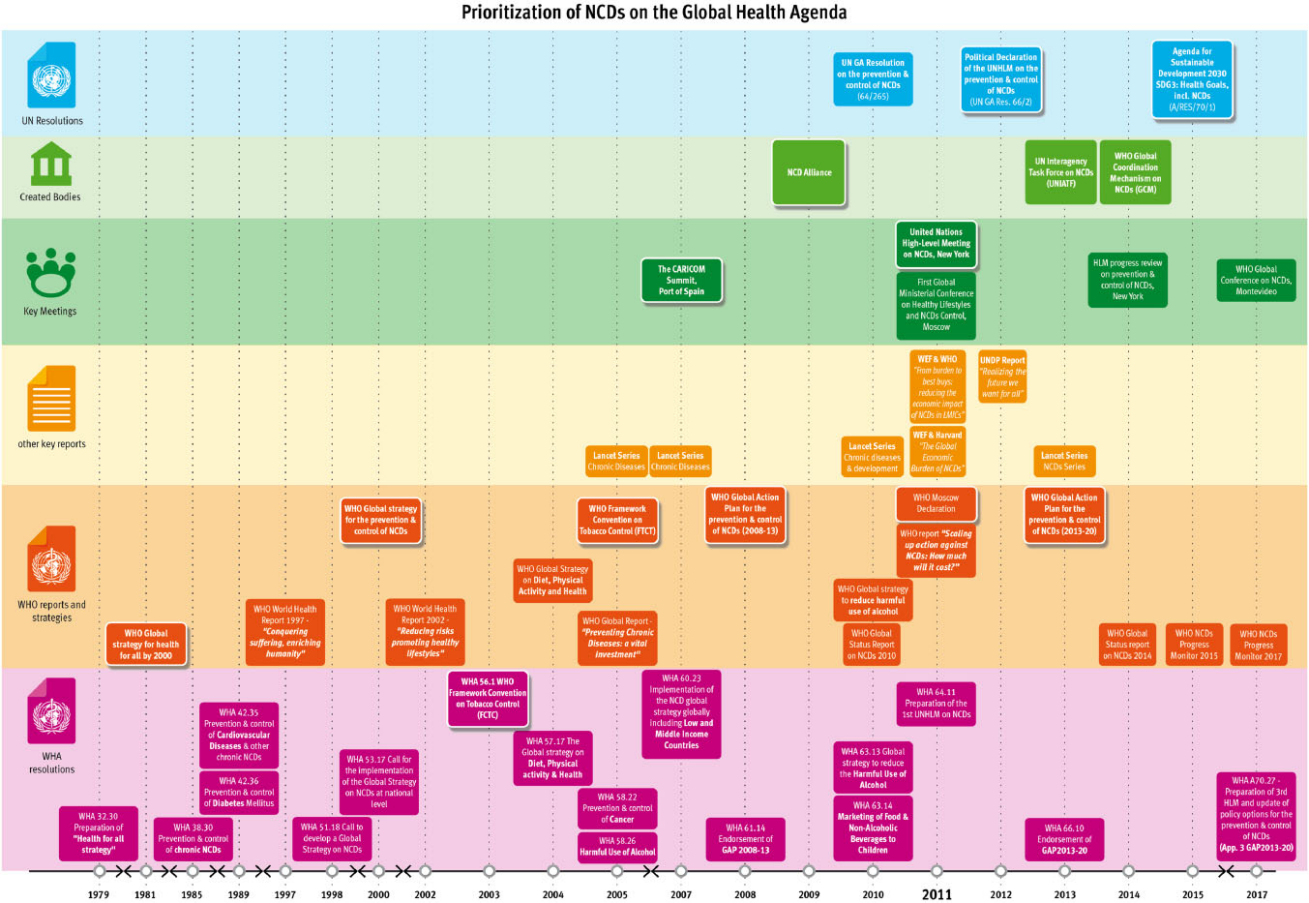
Timeline: prioritization of NCDs on the global health agenda.

The document review identified 48 key documents, among which 19 WHO reports, 6 academic publications and reports, 1 regional declaration (CARICOM), 17 WHA resolutions and 5 UN resolutions, published between 1981 and 2017. (Table 2) The timeline was complemented with findings from the interviews (Figure 2). Eleven semi-structured interviews (female = 5/male = 6) were conducted with individuals representing different sectors (WHO, civil society, private sector, academia and government). It is interesting to note that during the development process to reach the 2011 UNHLM and GAP, several of these individuals worked in different sectors as described in Table 3. Findings from both the document review and interviews are presented thematically, according to the Shiffman and Smith (2007) model with quotes from interviews and documents used to illustrate these themes.

**Table 2:** Key documents reviewed 1979–2017

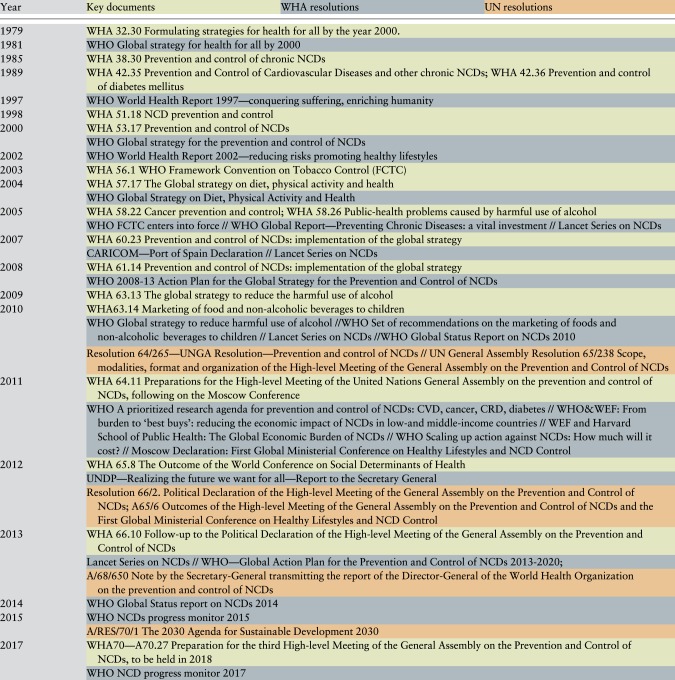

**Table 3:** Description of Interviewees by sector

	WHO	Civil society	Private sector	Academia	Government
I1		X			
I2		X			
I3	X	X			X
I4				X	
I5	X				
I6	X				
I7	X				
I8	X			X	
I9		X			
I10			X		
I11	X	x	x		

### Actor power

Actor Power is comprised of the strength of the individuals and organizations concerned with the issue relating to policy community cohesion, leadership, guiding institutions and civil society mobilization. Key actors were identified as major influencers in the NCD policy community, including individual champions and civil society, regional political voting blocs, leading institutions and academia.

Policy community cohesion is defined as the degree of unity between the various actors involved in the issue (Shiffman and Smith, 2007). While there may still be a lack of policy community cohesion, the geopolitical alignment of certain UN voting blocs was critical in gaining the necessary traction to move towards a UNHLM and political declaration for NCDs. Many of the interviewees described mini-coalitions of countries or other stakeholders that joined forces to get NCDs on the agenda.

*[…] the Scandinavian governments, Sweden, Denmark, Norway […] have always been very strong on NCDs. … Then you’ve also obviously got the […] BRICS (Brazil, Russia, India, China, South Africa) countries where the burden is the greatest, in terms of sheer numbers. … BRICS countries [are] beginning to take leadership role* (I1).

Interviewees from NGOs, private sector, academia and WHO all highlighted the leading role played by the Caribbean Community (CARICOM) and also Commonwealth countries:

*All the Caribbean countries got together. They all got together in 2007* (I2).

The Commonwealth countries had two political blocks:

*There’s the Caribbean and there are the Pacific Islands. Both those blocks, which when you put them all together, there you have considerable voting numbers in the UN* (I9).

Another cohesive group of actors was the NCD Alliance, created in 2009 by four disease-specific federations: International Diabetes Federation (IDF), World Heart Federation, Union for International Cancer Control and International Union Against Tuberculosis and Lung Disease.

*The reason why those four actors came together at that time was because they all recognized that they had […] gone as far as they could in terms of their own advocacy on disease specific issues, […] They also recognized […] a shared agenda around […] the shared risk factors and similar kind of health system response required* (I1).

Within the NCD policy community, various leaders advocating for NCDs and policy development were mentioned by the informants. Specifically, the leadership of the IDF was seen as:

*a major proponent, a major supporter of there being a UN-type meeting and the [CEO of IDF] also persuaded colleagues and the other three organizations […] that they should come together in an alliance and that alliance was then a major proposer for there to be international attention to NCDs* (I3).

Specific individuals from the Caribbean were mentioned by all interviewees as being:

*Always the kind of political champions of the NCD issues, particularly [at] the UN* (I1).

Academics were also seen as responsible for disseminating evidence on the NCD burden. This included four series of publications from the Lancet action group on NCDs (The Lancet, 2005, 2007, 2010, 2013). The role of the private sector was also highlighted by some of the interviewees and in the GAP’s overarching principles and approaches.

*It should be recognized that effective NCD prevention and control require leadership, coordinated multi stakeholder engagement for health both at government level and at the level of a wide range of actors,… with relevant civil society and private sector entities* (World Health Organization, 2013).

In terms of guiding institutions, interviewees suggested that, the technical lead was assumed by WHO, but that resulted in certain limitations. As stated by I5, ‘[WHO] is not really the global police. We are actually the Secretariat of the Member States’.

In order to expand the network of stakeholders involved and address some of its institutional constraints, including the involvement of non-state actors and especially the private sector, WHO established the Global Coordinating Mechanism (GCM) in 2014 (World Health Organization, 2017a) with the aim to ensure the implementation of the GAP by engaging WHO Member States, UN organizations and non-state actors. The premise of the GCM was cited in the GAP, as a ‘global mechanism to coordinate the activities of the UN system and promote engagement, international cooperation, collaboration and accountability among all stakeholders’ (World Health Organization, 2013). Some interviewees highlighted the limitation of the GAP:

*I don’t think what we’ve got at the global level is necessarily the right kind of architecture to be moving forward. I don’t necessarily think WHO is the one that will be able to do it. It’s just a question of how you facilitate a shift outside of WHO on these issues* (I1).

Much of the successful advocacy work to elevate the priority of NCDs interventions parallels the emergence of the NCD Alliance. However, civil society movements behind the four main diseases remain relatively fragmented and weak; as stated by I5:

*Unlike HIV or TB, a collective civil society presence has not come in NCDs.*

I7 adds, a ‘lack of social mobilization is [a hurdle], definitely’.

### Ideas

The Ideas category refers to the way that the issue is portrayed and understood by those involved (Shiffman and Smith, 2007). It is comprised of the internal frame, which seeks to grasp the level of consensus within the policy community, and the external frame which is the public portrayal of the issue. Identifying a set of ideas to attract political and public support has been a persistent challenge of NCDs prioritization. This has resulted in an evolving framing used to characterize NCDs, from focusing on morbidity and mortality to economic and development concerns, and finally to the adoption of a human rights approach.

The NCD policy community agreed in 2000 on the first framing of NCDs as ‘4 × 4’, four risk factors and four diseases.

*Four of the most prominent noncommunicable diseases—cardiovascular disease, cancer, chronic obstructive pulmonary disease and diabetes—are linked by common preventable risk factors related to lifestyle. These factors are tobacco use, unhealthy diet and physical inactivity* (World Health Organization, 2000a).

Several interviewees emphasized that this framing was needed to simplify the complexity of NCDs as a group of diseases but also stated:

The 4 × 4 isn't perfect, but it's what we've got (I9).

‘Best Buys’ for NCDs were developed as the solutions within the internal frame for the purpose of the external frame. This concept appeared in WHO Global Status Report 2010, as ‘cost-effective, feasible and affordable interventions in any resource setting’ (World Health Organization, 2011). In the lead up to the 2011 UNHLM, ‘Best Buys’ were included in several key documents, most importantly the 2011 World Economic Forum (WEF) reports and WHO report on scaling up action against NCDs (Bloom *et al.*, 2011; Chisholm *et al.*, 2011; World Economic Forum and Harvard School of Public Health, 2011). Subsequently, these were included in the updated Appendix 3 of the GAP during 2017 World Health Assembly (World Health Organization, 2017b).

The external framing of NCDs has used various ways of highlighting the importance of these diseases with security related, economic development, sustainable development, socio-economic development and finally human rights language used. Certain WHO policies and strategies have employed the urgency of security-related language. Framing NCDs as a threat to economic and human development was first used in WHA51.18 (World Health Organization, 1998):

*they [NCDs] cause enormous human suffering and threaten the economies of Member States, where costly treatment will further deprive the poor and powerless and increase the inequities in health between population groups and countries* (World Health Organization, 1998).

The 2000 Global Strategy described the ‘global threat’ posed by NCDs and the need to provide ‘urgent and effective public health responses’ (World Health Organization, 2000a). In 2005, WHO described NCDs as, ‘[…] an under-appreciated cause of poverty and hinders the economic development of many countries’ (World Health Organization, 2005). In 2007, WHA60.23 noted the ‘links between NCDs, development, the environment and human security,’ as well as their contribution to ‘health inequalities’ (World Health Organization, 2007). In 2008, the first action plan towards a global strategy for prevention and control of NCDs emphasized that addressing NCDs is ‘an integral part of sustainable socioeconomic development’ (World Health Organization, 2008). The 2011 UN Political Declaration focused on the social, economic and development impact of NCDs on countries, especially LMICs (UNGA, 2012). Currently, GAP highlights the work of WHO to ensure that the burden of NCDs does not ‘undermine the development gains of past years’, (World Health Organization, 2013). I1 provides an example of the different portrayal of NCDs:

*Everyone always throws around the figure of $47 trillion it's going to cost the world in the next two decades on NCDs. The economic case became a lot clearer, as did the kind of rights and social justice issues of NCDs. […]. I think that is a kind of re-framing of the issues, which I think helps catalyze physical commitment*.

Three challenges with regards to the external frame and the proposed solutions for NCDs were detailed by the informants. Firstly, the issue of prevention vs care:

*What I have tried to set up here is a framework that makes it possible for politicians to make policy options on especially the prevention side. The management side is only a kind of a consequence of not being able to prevent properly* (I7).

Secondly, the wider links between NCDs, development and human rights:

*A huge amount of push-back from a lot of the development community on NCDs being recognized as a development issue, despite the fact that [NCDs are now] in the SDGs* (I1).

Similarly, human rights language has difficulty emerging in the framing:

*[There is] still push-back in terms of recognizing that it's a poverty issue and a rights issue. They still claim that there's not enough evidence to prove that when there is* (I1).

I7 adds, ‘you could ask the same question on human rights. The only place in the global action plan where this is stated is in the overarching principles, and there is no operationalization of that’

Finally, how to provide policymakers with concrete solutions is difficult for NCDs, despite the existence of the ‘Best Buys’ as:

*We've never said that the 4* × *4 is transferable to every single country and every single community. It's obviously a bit like IKEA furniture. You know people need to tailor it and fiddle around until they get, that it makes sense to them in context* (I1).

### Political contexts

Political contexts are the overall environment in which the policy actors operate (Shiffman and Smith, 2007). Two elements characterize this category; policy windows and global governance structures. Policy windows provide actors with opportunities to influence decision-makers. While the global governance structure is the existence of a ‘platform’ allowing for ‘effective collective action’ (Shiffman and Smith, 2007). Although many policy windows for NCDs have occurred, the limitations in the global governance structure meant these windows did not yield the desired results. NCD policy windows have mainly been global events bringing together a variety of policymakers.

One policy window was a meeting in 2007 held by the CARICOM countries that was instrumental in that it called for a Resolution for a UNHLM and UN General Assembly support. The outcome document, The Port of Spain Declaration marked the first high-level political commitment to address NCDs, which was perceived to be ‘a watershed moment’ (I2).

Another key policy window was the UNHLM in 2011. The gravity of the situation had been grasped and ‘all the stars were in alignment’ (I3); particularly since the Port of Spain Declaration and the creation of the NCD Alliance in 2009. While the political declaration that came out of this meeting was not binding under international law, it had political weight and was significant in the lead up to the SDGs. In this context, the successful inclusion of NCDs within the SDGs (World Health Organization, 2017c) was seen as:

*… Even bigger [than the UNHLM], because then it's clearly stated that NCD is not only a health threat but also a development threat* (I10).

In terms of global governance, WHO prioritized NCDs as one of six clusters at the headquarters in 2018 (World Health Organization, 2018b). However, policies, strategies and resolutions continue to reflect the non-unified nature of different units or disease issues. For example, a number of WHA Resolutions and WHO documents are either specific to a given disease or risk factor (e.g. the Global Strategy on Diet, Physical Activity and Health or the Global Strategy to reduce the harmful use of alcohol) (World Health Organization, 2004, 2010) or propose a more general approach encompassing all NCDs and their risk factors (e.g. WHA53.17 and WHA60.23 and the two NCD global action plans) (World Health Organization, 2000b, 2007, 2008, 2013).

Interviewees noted that the GAP developed by WHO (World Health Organization, 2013) was the response to the commitments made by Heads of State and Governments in the UN Political Declaration (UNGA, 2012). This was seen as the main guiding document for the global NCD response. This document is used within the global governance structure of WHO which operates only in the area of health, and this was seen as needing to be expanded:

*We can only plead and advocate that the Ministry of Transport will reduce emissions, or the Ministry of Food to reduce sugar […] Ministries [of Health] are very weak. So, it all depends on how the heads of the governments take it up…* (I5).

Finally, I11 adds that:

*There is no whole-of-society-approach. … you need a more enlightened approach at WHO and amongst policymakers to get out of a narrow, a few policies alone will do it, into a broader, more creative mindset.*

To address the challenge of WHO’s limitations in working beyond the health sector, mechanisms were developed, such as GCM and the United Nations Interagency Task Force on the Prevention and Control of NCDs (UNIATF).

*[GCM] Terribly named thing … I don't think we've really cracked the nut, in terms of what works, in terms of multi-sectoral engagement. In terms of kind of the key players* (I1).

I1 adds:

*What we’re finding is that because [the GCM is] based in WHO it's fairly limited in terms of actually its ability to meaningfully engage with civil society and private sector* (I1).

One interviewee also recognized the positive development of UNIATF in having more actors around the table discussing NCDs.

*WHO is obviously the main technical agency, but now we’ve got a UN inter-agency taskforce on NCDs which brings together all sorts of different UN agencies around NCDs, which is an interesting development from our perspective because now you’ve got the World Bank, you’ve got UNICEF, you’ve got UNFPA, UNAIDS, all beginning to talk about NCDs* (I1).

### Issue characteristics

Issue characteristics are comprised of credible indicators, severity and effective interventions.

The GAP includes nine voluntary global targets, which aim to measure changes, mortality, risk factors and use and access to medicines (World Health Organization, 2013) (Table 4). Although identified as useful targets and objectives, informants question their applicability globally:

*[They] help mobilize and galvanize countries. As with any global plan getting […] to translate into national action; is not easy* (I2).

I1 adds about these targets:

*It's completely unrealistic and unfeasible to think that a low-income country is going to be able to do everything […] even setting itself all nine targets and 25 indicators, which is exactly the same as the global framework. It's just not possible* (I1).

**Table 4:** Global Action Plan voluntary targets for NCDs

1. A 25% relative reduction in risk of premature mortality from CVD, cancer, diabetes or CRD.
2. At least 10% relative reduction in the harmful use of alcohol, as appropriate, within the national context.
3. A 10% relative reduction in prevalence of insufficient physical activity
4. A 30% relative reduction in mean population intake of salt/sodium.
5. A 30% relative reduction in prevalence of current tobacco use in persons aged 15+ years.
6. A 25% relative reduction in the prevalence of raised blood pressure or contains the prevalence of raised blood pressure, according to national circumstances.
7. Halt the rise of diabetes and obesity.
8. At least 50% of eligible people receive drug therapy and counselling (including glycaemic control) to prevent heart attacks and strokes.
9. An 80% availability of the affordable basic technologies and essential medicines, including generics, required to treat major NCDs in both public and private facilities.

In terms of severity, there is widespread consensus that NCDs present an unprecedented and incomparable challenge to global and national health systems (Beaglehole *et al.*, 2011a). WHA resolutions in 1985 and 1989 already alert to the strong impact of NCDs (World Health Organization, 1985, 1989a, 1989b).

*Recognizing that diabetes mellitus is a chronic, debilitating and costly disease attended by severe complications including blindness and heart and kidney disease; Noting that diabetes already represents a significant burden on the public health services of Member States, and that the problem is growing, especially in developing countries* (World Health Organization, 1989b).

I8 stated that,

*There’s plenty of data out there. Not in the form that was available for consumption by policymakers and their advisors. It’s very interesting a lot of our messaging at WHO was still in very technical terms* (I8).

The different ways of presenting the severity of NCDs in WHO documents is included in Table 5.

**Table 5.** NCDs severity presented in WHO documents reviewed

WHO document	Presentation of severity
Global Strategy for the Prevention and Control of NCDs, 2000 (World Health Organization, 2000a)	NCDs were responsible for 60% in 1998 (or 31.7 million) deaths annually, and represented 43% of the global burden of disease
Preventing chronic diseases: a vital investment, 2005 (World Health Organization, 2005)	Chronic disease will account for 35 million deaths in 2005, which is double the number of deaths from all infectious diseases (including HIV/AIDS, tuberculosis and malaria), maternal and perinatal conditions and nutritional deficiencies combined
2008–13 Action Plan for global strategy for the prevention and control of NCDs, 2008 (World Health Organization, 2008)	NCDs are growing to dominate healthcare needs in LMICs and that by 2013 these countries were already bearing 86% of the burden of NCD-related deaths
Global status report on NCDs 2010, 2011 (World Health Organization, 2011)	Need to launch a more forceful response to the growing threat posed by NCDs.Particular attention is given to conditions in LMICs, which now bear nearly 80% of the burden from NCDs.
From Burden to ‘Best Buys’: Reducing the Economic Impact of Non-communicable diseases in LMICs, 2011 (World Economic Forum and Harvard School of Public Health, 2011)	Cumulative economic losses to LMICs from the four core NCDs will exceed USD 7 trillion between 2011 and 2025
The Global Economic Burden of NCDs, 2011 (Bloom *et al.*, 2011)	Over the following 20 years, NCDs would cost more than US$ 30 trillion, which represented 48% of global GDP in 2010, and would push millions of people below the poverty line
Global action plan for prevention and control of NCDs 2013–20, 2013 (World Health Organization, 2013)	63% of global deaths

Informants also cited the essential role of academic publications in supporting the severity case of the issue in terms of ‘high mortality, morbidity, or socioeconomic cost’. I8 and I3 both discussed a variety of publications in academic literature that provide substantive evidence of the scale of the NCD challenge ahead:

*Th[e] [Lancet Series 2005] was sort of making the academic case* (I8) and *pointing out to the gravity of the problem and the need for there to be a concerted, international attention* (I3).

Both 2010 and 2014 global status reports on NCDs highlighted the availability of effective interventions with evidence of clear and measurable impact exemplified by specific case studies (World Health Organization, 2011, 2014). Policy documents such as the World Economic Forum (WEF)-WHO joint paper on ‘Best Buys’ focused on demonstrating the cost-effectiveness of policy response options, which have proven to be effective in different contexts (World Economic Forum and Harvard School of Public Health, 2011). However, the research shows that while tools are available, implementation remains an issue:

*Most of the interventions in low-income countries, and some in high-income countries are not working, whether it is a regular cancer screening or hypertension control or management of diabetes* (I5).

The interventions described in the GAP include a list of options that, while not exhaustive, are intended to provide information and guidance on effectiveness and cost-effectiveness of interventions based on current evidence. Additionally, it is stated that when selecting interventions, consideration should be given to national circumstances (World Health Organization, 2013). Interviewees also highlight that interventions for NCDs are complex:

*I always say ‘what is the equivalent of a condom in diabetes?’ It is not there. HIV, we can go to you in assembly and have a condom in our hand and say*, *‘this is it, use this, make this available, you control the disease’* (I5).

Added to this complexity is the time lag between action and outcome. I1 describes this as:

*You’re not going to see very much impact if you’re going to be investing in things like childhood obesity and tobacco control a little bit more, alcohol control, these things take a long time. It’s to do with behavior change. It’s to do with changing the environments that people are living in to promote healthy options and that there’s no kind of magic bullet so kind of incentivizing funding into prevention, I think, is inherently very difficult.*

Given the underlying social, economic and political factors of NCD risk factors, proposed interventions may not be perceived as politically appealing in all contexts. Interviewees clearly express this limitation.

*You have the commitments, you have the tools, you know how to measure, you measure it, you know how to hold people accountable and still there is so little happening* (I7).

This is compounded by two factors mentioned by I7 and I10: the need for multi-sectorial plans and the issue of lack of funding at both global and national levels.

*Even if we have had more and more countries coming up with national plans they are not really multi-sectoral and they are not financed and they are not budgeted, they are not prioritized* (I7).

### Linkages between the different elements

The overall linkages between the different elements from Shiffman and Smith framework detailed above are presented in Table 6. This shows how the linkages between the different elements of the Shiffman framework can be seen as bi-directional, with for actor power being influenced by ideas and ideas influenced by the actors. No single factor is sufficient to explain the relative lack of priority afforded to these NCDs as a category over the past few decades, nor their more recent rise on the global agenda.

**Table 6:** Bi-directional Linkages between different elements of the Shiffman and Smith (2007) framework for NCDs

Bi-directional linkages between	Actor power	Ideas	Political contexts	Issue characteristics
Actor power	Groups of countries and organizations came together on the issue of NCDs (e.g. CARICOM; disease-related organizations) Leaders exist, but these are technical insiders vs people able to transcend the global health community WHO is the main guiding institution, but it is not its role to enforce certain measures especially with NCDs where areas beyond heath need to be addressed. The GCM and UNIATF were established to counter some limitations of WHO, but not effective enough at multi-sectorial engagement Civil society is weak and mainly being led by the NCD Alliance. The organizations involved in the Alliance are not truly grassroots organizations and were brought together with the 4 × 4 framing.	Actor power created the internal frame—4 × 4—grouping of NCDs into one disease category improved traction in their prioritization globally Actors being mainly in health face challenge to get their message across to non-health audiences	Actors have pushed to create policy windows—the geopolitical alignment was critical in gaining the necessary traction in order to move towards a UN high-level meeting (UNHLM) and political declaration for NCDs. The policy agenda which was in the realm of the WHO needed to be addressed at the UN for the UN then to give the mandate back to WHO Civil society effectively used the political grouping of diseases and economic argument to attract political attention. The network of NCD experts have not successfully expanded their political coalition from the realm of health to other stakeholders whose engagement is required The WHO lacks the authority to steer the NCD issue and expand the stakeholders engaged with NCDs interventions, particularly beyond traditional health actors.	Health Actors have influenced the indicators used, which primarily stayed in the realm of health. Many of these focus on prevention vs treatment
Ideas	High agreement on NCD challenge within NCD community with significant evidence on its burden. The NCD policy community agreed in 2000 on the first framing of NCDs as ‘4 × 4’, four risk factors and four diseases. In order to address these different audiences, the framing of NCDs shifted from morbidity and mortality to economic and development concerns.	Framing and language is not effective beyond health experts Complexity of issue cannot be simplified Bringing together of four diseases may make sense from a health perspective, but again might be a challenge from a framing perspective as these diseases are very different	NCDs encompass such a wide range of factors, difficult to know where to start for policymakers (prevention vs care) Identifying a set of ideas to attract political and public support has been a persistent challenge of NCD prioritization and this has resulted in evolving framings used to characterize NCDs.	The framing of NCDs has evolved starting with morbidity and mortality then evolving to economic development concerns Certain WHO policies and strategies have employed the urgency of security-related language to describe the need to provide urgent and effective public health responses. Effective solutions or best buys include the need for involving other sectors, e.g. taxation issues
Political contexts	The Port of Spain Declaration marked the first high-level political commitment to address NCDs. Advocates have sought the inclusion of NCDs on global meetings in order to build a favourable political environment and take advantage of policy windows UNHLM was a policy window which provided actors with opportunities to influence decision-makers NCD issue taken to UN to highlight political need for action from various actors (beyond health), but the responsibility is sent back on WHO. This has not resulted in any increase in actors involved in NCDs or leaders outside the health arena to be identified	Political context influenced the framing development—the choice of framing had the aim to trigger political traction.	Policy windows have been present, but no tangible results from these. There is a lack of a strong global governance structure able to cope with the complexity of NCDs and the need for action beyond the health sector. While the political declaration that came out of it was not binding under international law, it had political weight and was significant in the lead up to the SDGs. WHO’s limitations in working beyond the health sector, mechanisms were developed to get different areas working together, such as GCM and UNIATF	NCDs encompass such a wide range of issues requiring both health and non-health issues thus the indicators although health related require wider buy-in from other sectors Issues around whether or not proposed interventions are adapted and realistic for implementation especially in LMICs To date no funding has been allocated to address this effectively
Issue characteristics	Academic publications played an essential role in supporting the severity case of the issue in terms of “high mortality, morbidity, or socioeconomic cost”.	The economic argument presenting rising costs and burden was key to advance the global health policy response	Effective interventions exist (“Best Buys”), but are complex and require a multi-sectoral approach with political commitment. Severity of NCDs are presented in a variety of ways: deaths and economic burden, but issue is with translation to something useful for policymakers	Credible indicators exist and can be measured, but progress is hard to measure and validity of some indicators is questioned as are their relevance for countries

Even though the emergence of NCDs as a political category of diseases can be identified as early as the mid-1980s, recent years have witnessed more documents, meetings and attention for NCDs (Figure 2) with certain individuals being key in driving the NCD policy agenda. The network behind NCDs is predominantly confined within the health sector, with this leadership from health experts shaping both the framing of the issue, as well as proposed solutions.

The grouping of NCDs and their associated risk factors (4 × 4 framing) had an impact on both the issue characteristics and actor power. For issue characteristics, this combination of the four NCDs leveraged the severity and collective impact. In actor power, this consolidated and strengthened the policy community cohesion, by creating a larger pool of leaders to champion NCDs, and enabled the institutional prioritization of NCDs as a focus area of the WHO. In parallel, this strengthened civil society engagement through the creation of the NCD Alliance.

NCD advocates have been successful in creating policy windows such as the UNHLMs or the inclusion of NCDs in the SDGs. However, political decisions resulting from these opportunities have yet to materialize in substantive ways. This is in part due to limitation of the proposed solutions not being adapted to different contexts given the current global governance structure and actors involved. WHO being central to global governance structure in moving the NCD agenda forward is limited in the extent to which it can engage other actors beyond Ministries of Health on this issue.

The CARICOM countries and their leading role show the importance of the associations between the elements of the Shiffman and Smith framework. The CARICOM’s role was based on the following factors: unified voice of a group of countries (guiding institutions); involvement of certain individuals playing multiple roles at national and regional levels (leadership); inclusion of experts from different sectors in the discussions which helped reframe the issue away from one purely focused on health (ideas and severity); the Port of Spain meeting (policy window) was effectively used to garner support from the CARICOM governments; and the CARICOM countries within the UN system carry many votes (global governance structure). All these elements translated into the issue being raised at the UN by the CARICOM countries with their representatives at 2011 UNHLM pushing for this agenda (leadership and global governance structure).

## Discussion

This study highlights how institutions and leaders from the actor power component were able to leverage existing evidence and use this for action by framing NCDs in a more comprehensive way. Two framings were essential for NCDs to gain traction on the UN health agenda. Firstly, the simplification of this disease category through the 4 × 4 framing, and secondly, the shift away from presenting NCDs in terms of morbidity and mortality towards focusing on an economic argument which highlighted the development challenges. Civil society was also able to effectively use the political grouping of diseases and economic argument to attract political attention.

Community cohesion through CARICOM, NCD Alliance and certain key leaders was instrumental in getting the UNHLM in 2011. These actors recognized that in order to move the NCD response forward, the issue had to be politicized beyond the scope of WHO to the wider attention of the UN. The UN was then able to reinforce WHO’s mandate of institutional leadership on the global NCDs agenda. The inclusion of NCDs within the SDGs is possibly the largest opportunity for the issue to be included in global and national responses as well as linked to wider issues included in the SDGs, e.g. poverty, food security, education, etc.

Despite this progress, several issues prevail. These include the need to further expand the NCD political coalition from the realm of health to other stakeholders whose engagement is required to make progress. WHO’s role is to shape global and national policymaking, and its circle of influence is most often limited to health ministries (Magnusson, 2010). The proposed interventions not only require a wider implication of non-health actors, but also touch upon complex issues from prevention to treatment. The UNIATF and GCM are an opportunity to address these wider issues, but have yet to truly accomplish this. In terms of framing some have called for NCDs to be reframed as a threat to global security (Saha and Alleyne, 2018). This argument is based on the rationale that health issues which transcend nation state boundaries and pose a threat in terms of health security often receive rapid prioritization, global attention and funds (Horton, 2017).

In addition, there has been a relative lack of social mobilization around a comprehensive strategy against NCDs, and civil society actors often remained divided and failed to become true advocates for NCDs (Horton, 2015). This may be in part due to the challenges of the competing components of the NCD category, which is broadly conceived with the notable absence of mental health within the 4 × 4 category (Ngo *et al.*, 2013). This was recently rectified during the 2018 UNHLM (World Health Organization, 2018b) where mental health and air pollution were added. Another possible reason is that the network of civil society for NCDs is comprised of individual organizations dedicated to promoting progress against a specific disease, or set of diseases, instead of ‘NCDs’ as a whole.

The methodology used in this study is similar to other studies that have used the Shiffman and Smith framework as a mean to analyse different health issues (Shiffman and Smith, 2007; Shawar *et al.*, 2015; Berlan, 2016; Gneiting, 2016; Quissell and Walt, 2016; Schmitz, 2016; Shawar and Crane, 2017). The use of this framework strengthens the results by focusing on factors already shown to be significant in other areas of global health prioritization (Shiffman and Smith, 2007). The use of WHO GAP as a starting point is a limitation as this puts a certain focus on documents developed by WHO. A further limitation includes our access to and inclusion of only publicly available documents. As with any qualitative study, issues of bias exist, but the involvement of a multi-disciplinary team aimed to mitigate this both at the time of the interviews and during analysis of the data. Limitations include the date of the interviews and document review; interviews were carried out in 2017 and the document review does not include more recent documents related to the UNHLM in 2018 and the recent report of WHO Independent High-Level Commission on NCDs (World Health Organization, 2018c). However, given that this study covers key elements of the NCD policy agenda as well as being the first time to our knowledge that a comprehensive analysis of how this group of diseases gained attention in the UN system, it provides a clear contribution to the literature. Our interviews were also with individuals actively involved in the overall process of NCDs policymaking within WHO, thus limiting the view on the issue. That said the central role of WHO as well as small group of individuals in placing NCDs on the agenda is a key finding from this article and as discussed, a limitation on why the NCD agenda has not progressed. Using snowball sampling possibly biased the individuals included in this study, however, the interviewees included are from a range of different sectors.

It is proposed that the NCD response would benefit from ‘splitting’ the issue in two, with on the one hand a focus on risk factors and on the other access to treatment and care. The UNLM 2011 Declaration and GAP (UNGA, 2012; World Health Organization, 2013) have mostly focused on prevention while the absence of access to treatment and care is notably absent from policy and responses, with only two out of the eight targets in the GAP addressing this issue.

Risk factors for NCDs are multi-dimensional comprising biological, social, behavioural, economic and environmental factors. This limits the impact that any single intervention can make. The Framework Convention on Tobacco Control (FCTC) is a unique global health response in that it was the only time WHO used its role to negotiate an international treaty which resulted in binding international commitments (Wipfli, 2016) and allowed a specific governance mechanism to be developed for addressing one of the main NCD risk factors (Gneiting, 2016). This could serve as a model for other risk factors, even if these are more complex to deal with, and that for tobacco there was a certain ‘exceptionalism’ (Collin, 2012) due to its undisputed harmful nature.

The inclusion of NCDs and Universal Health Coverage (UHC) in the SDGs provides a unique opportunity for framing the issue of access to NCD treatments within a development, poverty and human rights perspective, as was the case for HIV/AIDS (Enoch and Piot, 2017). For treatment-related aspects, different actors need to be involved than for addressing the risk factors, evidence needs to be differentiated with local action prioritized over global co-ordination. Insofar as the pharmaceutical industry has shown some positive contributions to improve access to medications in other disease areas, engagement with the private sector will be necessary (Beran *et al.*, 2017). In parallel wider issues of strengthening health systems focusing on Primary Health Care are needed (Beran *et al.*, 2016).

Nishtar, WHO Independent High-Level Commission Chair, recently stated, ‘The good news on NCDs is that policymakers have both an awareness of the problem and an appetite for change. Unfortunately, this is not paralleled with action’ (Nishtar, 2017). This comment is supported by WHO’s NCD progress monitor showing that progress around the world has been uneven and insufficient, highlighting the need for bolder political action including the mobilization of domestic and external resources and safeguarding communities from interference by powerful economic operators (e.g. Tobacco and sugar industry) (World Health Organization, 2017d). Although the third UNHLM on NCDs in 2018 could have been seen as another policy window to further the global response for NCDs, the political declaration was described as ‘vague and unambitious’ (NCD Alliance, 2018). Recent changes in the structure at WHO have relegated the importance of NCDs with the disappearance of a specific Assistant Director General responsible for NCDs (World Health Organization, 2019); however, the high-level commission on NCDs (World Health Organization, 2019b) offers the opportunity of new leadership on NCDs albeit with a mandate ending this year. A real civil society movement and developing a global governance structure similar to the FCTC is warranted for the prevention-related agenda. With regards to access to care and medicines the overarching UHC agenda, also part of the SDGs, and national solutions need to be found, funded and implemented.

## Conclusion

NCDs represent a formidable policy and governance challenge for the global health community. The above analysis highlights three key lessons. Detailing the wide-ranging impact of NCDs was enabled through actors coming together and developing an evolving frame for these conditions by not only focusing on health-related factors, four diseases and risk factors, but also the economic and development impact arguments. Secondly, the nature of NCDs and associated risk factors are complex and inter-sectoral and require leadership, civil society mobilization and a coalition beyond the health sector. To date the proposed ‘Best Buys’ have limitations in their applicability, both at global and national levels, as well as lacking an effective mechanism for implementation and commitments. The SDGs offer the NCD community an opportunity as they not only include NCD specific targets, but also wide-ranging factors relevant to addressing this health challenge. Overall, there is the need for the NCD community to divide the issue into two components, prevention and treatment and care, as these require a different policy response at global and national level to ‘ensure healthy lives and promote well-being for all at all ages’.

## Ethical approval

This study was approved by the Commission Cantonale d'éthique de la recherche Genève in 2016.
